# Elevated compositional change in plant assemblages linked to invasion

**DOI:** 10.1098/rspb.2022.2450

**Published:** 2023-05-10

**Authors:** Alessandra R. Kortz, Faye Moyes, Vânia R. Pivello, Petr Pyšek, Maria Dornelas, Piero Visconti, Anne E. Magurran

**Affiliations:** ^1^ Department of Invasion Ecology, Institute of Botany, Czech Academy of Sciences, Průhonice CZ-25243, Czech Republic; ^2^ Biodiversity and Natural Resources Program, Biodiversity, Ecology and Conservation group, International Institute for Applied Systems Analysis (IIASA), Schlossplatz 1, Laxenburg 2361, Austria; ^3^ Centre for Biological Diversity, School of Biology, University of St Andrews, Fife KY16 9TH, UK; ^4^ LEPaC, Ecology Department—IB, Universidade de São Paulo, Rua do Matão, Travessa 14, São Paulo, SP CEP 05508-090, Brazil; ^5^ Department of Ecology, Faculty of Science, Charles University, Viničná 7, Prague CZ-12844, Czech Republic

**Keywords:** biological invasion, biodiversity change, global, invasive species, species replacement, turnover

## Abstract

Alien species are widely linked to biodiversity change, but the extent to which they are associated with the reshaping of ecological communities is not well understood. One possible mechanism is that assemblages where alien species are found exhibit elevated temporal turnover. To test this, we identified assemblages of vascular plants in the BioTIME database for those assemblages in which alien species are either present or absent and used the Jaccard measure to compute compositional dissimilarity between consecutive censuses. We found that, although alien species are typically rare in invaded assemblages, their presence is associated with an increase in the average rate of compositional change. These differences in compositional change between invaded and uninvaded assemblages are not linked to differences in species richness but rather to species replacement (turnover). Rapid compositional restructuring of assemblages is a major contributor to biodiversity change, and as such, our results suggest a role for alien species in bringing this about.

## 1. Background

Rapid compositional reorganization (temporal beta diversity) is a major contributor to the unprecedented rates of biodiversity change that characterize the Anthropocene (e.g. [1–5]). Alien species, considered one of the main five drivers of biodiversity change [6], are thought to be an important player in the process of biodiversity change via their effects on compositional reorganization [7–9]. There is support for the notion that alien species contribute to accelerating compositional change (e.g. [10,11]). However, the extent to which the presence of alien species is associated with short-term compositional change remains unclear.

Alien species have the potential to mediate biodiversity change through a number of different mechanisms, including the breakdown of biogeographical barriers [12], impact on native species richness and abundance as well as alteration of habitat structure, [7,8,13–17]. Alien species, especially when they become invasive, can impact native species in several complex ways, such as by shifting native species richness and abundance [7,8,18,19], reducing their fitness and behavioural activity [20] and changing plant-flower visitor networks [21]. Alien species have been identified as the second most important threat leading to extinction (e.g. [22–25]). Invasions can also change ecosystem functioning and thus jeopardize human livelihoods [26]. These influences can be subtle, can occur at different spatial scales [27,28] and may arise through interaction with different drivers. Ecological assemblages are not static entities. Indeed, as MacArthur and Wilson observed [29], the composition of all assemblages will vary through time as a consequence of the balance between immigration and local extinction. A first step, therefore, is documenting the characteristics of temporal compositional change in assemblages with and without aliens.

Recent research suggests that temporal beta diversity is much stronger for aliens than native arthropod species [30]. A recent global meta-analysis has further revealed that alien species drive biotic homogenization, but that this is dependent on the realm, being stronger in marine and freshwater systems than in terrestrial ones [31]. Thus, although the importance of invasions in contributing to compositional reorganization has been recognized [9,11,16,32–34], it is challenging to quantify the extent to which it is elevated relative to background rates.

Here we address this challenge by examining the vascular plant assemblages available in BioTIME [35], which is the largest public biodiversity time series database in the world. BioTIME is not specifically designed for alien species, but because it contains information on all species recorded in each site, it is possible to match the species with relevant databases on alien species to identify which sites contain alien species. We used the Global Naturalized Alien Flora database (GloNAF) [36], the most up to date global database on the distribution of naturalized alien plants in the world, to identify alien plant species in each region (i.e. country or state), and divided local assemblages into those in which alien species have been recorded (at any point during the time series) (with aliens) and those where alien species have never been detected (no aliens). Due to the effects of alien species on compositional reorganization, our expectation is that assemblages that contain aliens will exhibit higher levels of temporal beta diversity than uninvaded ones but this increase will not necessarily be reflected in higher levels of temporal alpha diversity. Analyses of time series data may provide more direct estimates of biodiversity change, in terms of temporal alpha diversity (number of species and rank abundance curve) as well as temporal beta diversity (compositional change or dissimilarity indices). For instance, changes in rank are related to switching of species abundances within the assemblages [37]—which is particularly important in the context of biological invasions, in which alien species themselves can increase their abundances over time. Specifically, components of temporal beta diversity allow us to pinpoint whether invasions cause a decrease in richness, changes in composition or both.

Given the rapidity with which compositional reorganization can occur [38], we quantified compositional change between successive census dates. We further partitioned this compositional reorganization into change resulting from a shift in richness (nestedness) and change due to species replacement (turnover) [39,40]. Since the turnover component predominates in most partitions of temporal compositional change [38], we expect it to account for the largest fraction of dissimilarity in these assemblages. To place our temporal beta diversity analysis in context, we additionally examine temporal alpha diversity and rank shifts. Our focus on plants recognizes their importance in contributing to the diversity and function of terrestrial ecosystems (e.g. [41–43]).

## 2. Methods

Here we investigated the vascular plants time series that are available in the BioTIME database [35,44]. BioTIME collates assemblage-level abundance data collected in a consistent way through time.

**Figure 1.**
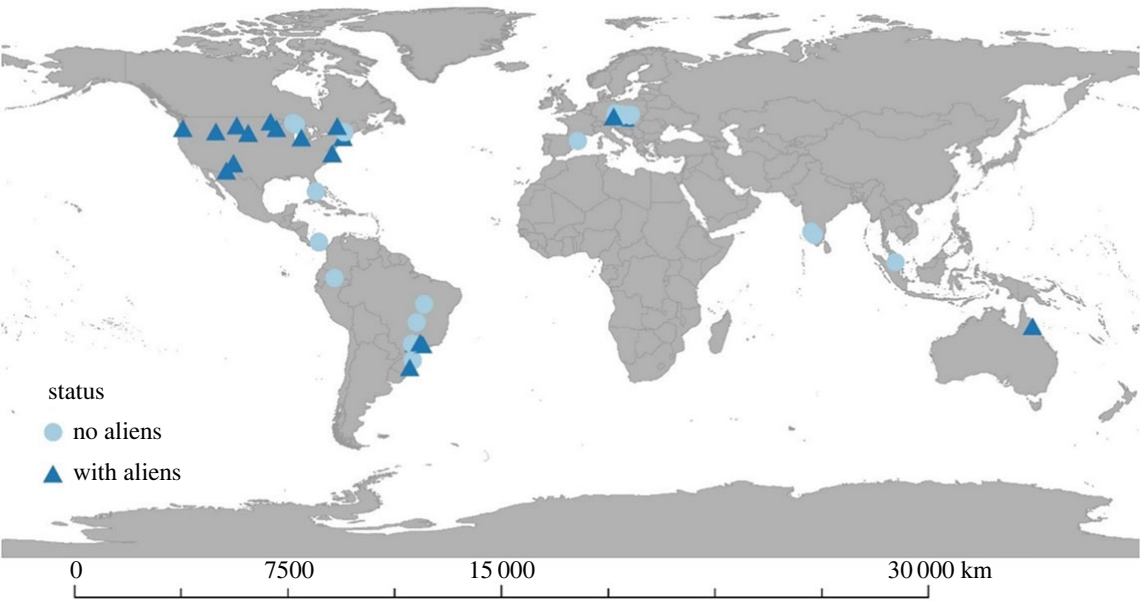
Vascular plant assemblages in the BioTIME database analysed in this study. Assemblages with alien species are denoted with a triangle symbol and those without alien species with a circle.

We selected individual studies that have been sampled in at least 3 (not necessarily consecutive) years and focused on the studies with numerical abundance data (thus studies that have only biomass data available were not included). In total, these represent 184 875 records of vascular plants encompassing 3224 species, in 52 individual studies (referred to as assemblages) sampled in 11 countries with a temporal span from 3 to 41 years; these comprise data collected from 1910 up to 2016 (figure 1).

First, to make it possible to compare species names mentioned in BioTIME with species names in GloNAF, all original species names in our dataset were standardized using The Plant List [45] alongside the taxonstand package from R [46,47]. Second, to identify which species are alien in a specified region (state or country where the sampling occurred), we used GloNAF [36]. GloNAF is the largest and most up-to-date database of the global distribution of naturalized alien species.

For each assemblage, we noted the sampling effort per year (e.g. number of sampled plots). To ensure that computed diversity metrics were comparable over time, for the assemblages in which sampling effort differed among years, we employed sample-based rarefaction [3,48]. To do this, we randomly selected the same number of samples in each year in the time series, with this number matching the minimum number of samples found in any year. Thus, the data extracted from BioTIME were first curated considering species names and taxonomic harmonization, and sampling effort, and the origin of each species in the sampled region was then confirmed (native versus alien). Relevant environmental variables for each assemblage are summarized in the electronic supplementary material, table S1.

To quantify compositional change (beta diversity), we calculated Jaccard dissimilarity and then partitioned it into turnover and nestedness using the R betapart package [39,49]. We focused on the compositional change between each sequential time step (i.e. we computed dissimilarity between pairs of consecutive years). Next, we computed the median value of these metrics (to produce one summary value of each metric per assemblage). In this analysis, turnover quantifies species replacement, whereas nestedness captures changes in dissimilarity due to species richness (i.e. where the year with the lowest richness represents a subset of the richest year). Jaccard dissimilarity uses information on the presence and absence of species.

In each case, we compared metrics for sites with aliens versus sites without aliens. To account for geographical distance between assemblages in these tests, we ran GLM models adding latitude and longitude as random effects using the glm function from R [50].

To provide additional insights into any relationship between compositional change metrics and geographical distance, we examined distance decay plots. We also asked if we could reject the null hypothesis of no spatial autocorrelation in the compositional change responses of invaded and uninvaded assemblages using the ‘Moran.I’ function from the ape package in R [51].

Exploratory analyses revealed no significant trends in annual rates of the metrics we considered (observed species richness, differences in species richness, differences in species rank, total Jaccard, turnover and nestedness). This supported our approach of calculating and reporting the median value of these metrics (see electronic supplementary material, figures S3–S14 in the electronic supplementary material and electronic supplementary material, table S2 for more details). To understand if there were differences in species richness between sites with and without aliens, we computed the median number of species per assemblage across all time steps in each assemblage (thus reporting one value per assemblage). We also calculated the species richness difference between pairs of consecutive time steps within each assemblage, both for sites with, and sites without aliens. To do this, we used the RAC_difference function in the codyn package from R [52,53]. This RAC_difference metric computes the total number of species at time A minus the total number of species at time B divided by the total number of species shared between both sites. As such, species richness difference is bounded between −1 and 1 with 0 representing no change. Finally, we computed differences in species rank reordering using the RAC_difference function in codyn. As before, we report the median value of the results for the pairs of consecutive timesteps only. Finally, to test whether change between consecutive time steps accelerated over time, we performed Spearman tests (using the R ggpubr package [54]). Here we only considered assemblages with at least four time points.

## 3. Results

**Figure 2.**
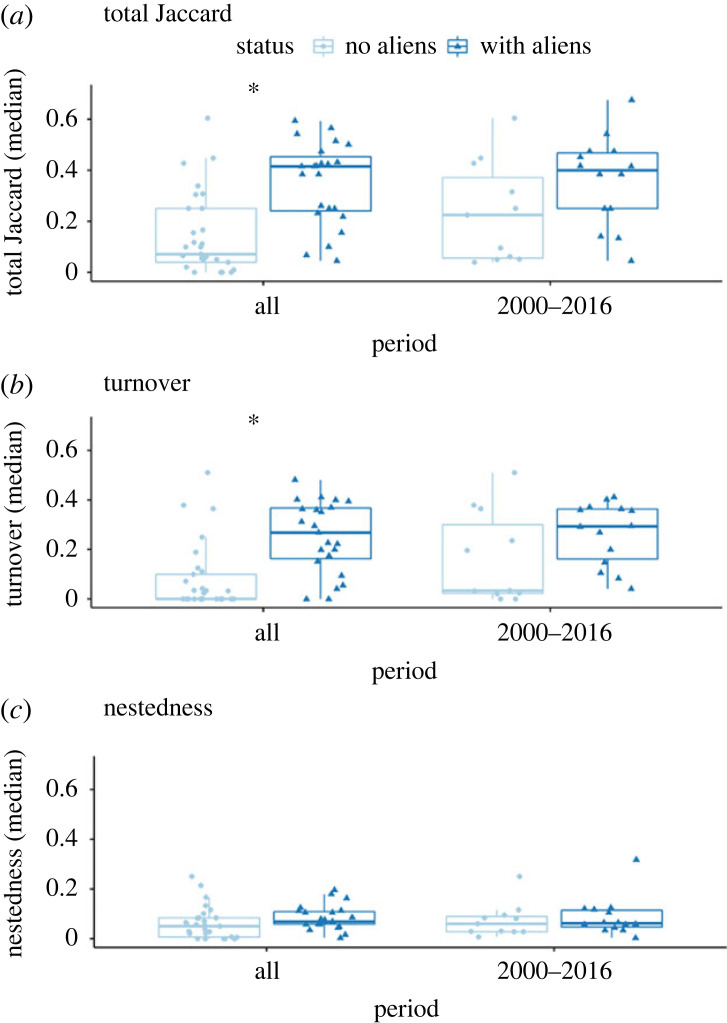
(*a*) Jaccard dissimilarity between time steps in assemblages with alien species (triangles) and without aliens (circles) for both all evaluated periods and for a subset of assemblages sampled between 2000 and 2016. Total Jaccard dissimilarity is partitioned into (*b*) turnover and (*c*) nestedness. Both total Jaccard and turnover are higher in areas with than without aliens for the entire period (see electronic supplementary material, table S4 for full summaries of the models).

Our analysis found that for both Jaccard dissimilarity and turnover, there was a significantly positive effect of an assemblage having aliens (JD *p* = 0.005; figure 2*a;* turnover *p* = 0.005; figure 2*b*). In neither case were the latitude and longitude effects significant. With nestedness, the effect of alien versus non-alien was also positive but non-significant (*p* = 0.48; figure 2*c*). However, in this case, there was a negative effect of longitude (*p* = 0.015; see electronic supplementary material, table S4 for full summaries).

Distance decay plots uncovered no significant relationship between site pairwise comparisons and geographical distance for all three metrics (electronic supplementary material, figure S1). Moran's I detected no spatial autocorrelation of Jaccard, turnover and nestedness in invaded and uninvaded assemblages (Moran's I output in electronic supplementary material, table S3). We did, however, find evidence of spatial autocorrelation in the full set of studies (invaded + uninvaded) for Jaccard and turnover (electronic supplementary material, table S2). In other words, when considering all assemblages together Jaccard and turnover values were more different in sites that were further apart. Despite this, there was no overall geographical bias between invaded and uninvaded assemblages (electronic supplementary material, figure S2).

**Figure 3.**
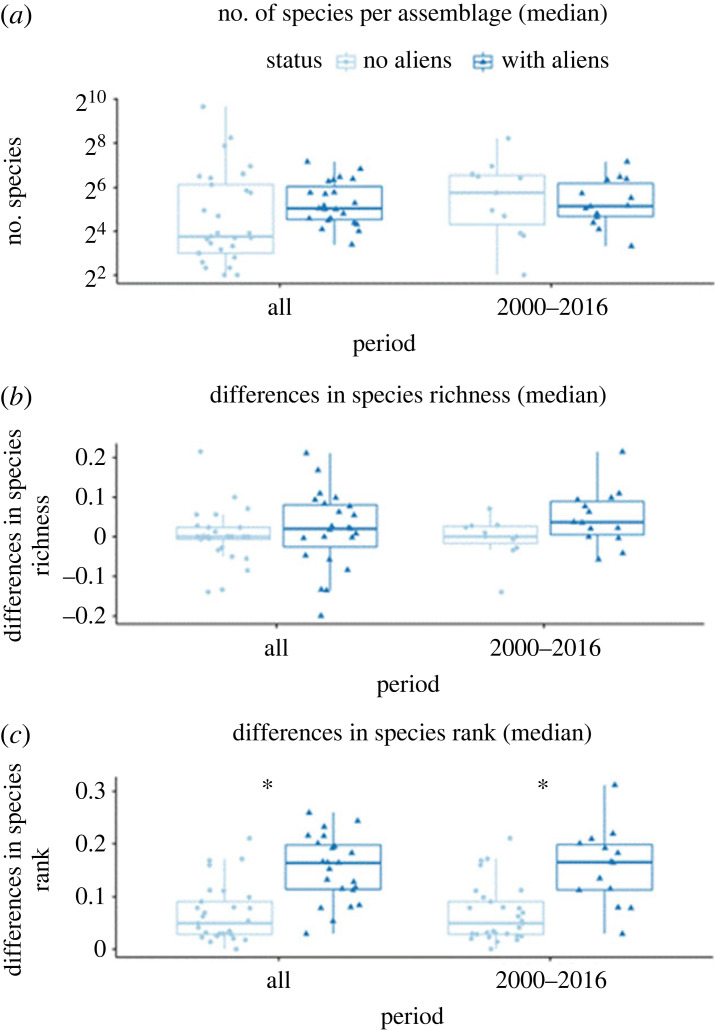
(*a*) Number of species sampled in each assemblage (median value among all sampled time steps in each assemblage, thus only a single value for each assemblage), in both areas with aliens and areas without aliens (note the *y*-axis is in log scale). In the entire period, median species richness was 13.5 in sites without aliens and 33 in sites with aliens; in the period between 2000 and 2016, median species richness was 55 in sites without aliens and 35.5 in site with aliens. (*b*) Differences in species richness and (*c*) differences in species rank (median value considering each consecutive time step, thus only one value per assemblage), both calculated using the RAC_difference function of the codyn package from R. In (*b*,*c*), only pairs of consecutive years are considered. There were differences in species rank in invaded compared to uninvaded assemblages (in both periods) (see electronic supplementary material, table S5 for full summaries of the models).

Most assemblages did not show a trend in consecutive year change (electronic supplementary material, table S2 and figures S3–S14). We detected, however, that some assemblages show faster rates of year-to-year compositional change (e.g. assemblages 356 and 465 in the electronic supplementary material, figure S10). However, there was broad consistency in the assemblage sizes, expressed as the numbers of species (figure 3). In other words, we found no significant differences in median species richness between invaded and uninvaded assemblages (figure 3*a*; *p* = 0.63; see electronic supplementary material, table S5 for full summaries of the models). Similarly, the difference in species richness between invaded and uninvaded assemblages was indistinguishable for the entire period (figure 3*b*; *p* = 0.22; electronic supplementary material, table S5). At the same time, invaded sites had higher rates of change in species rank—shuffling of species abundances (figure 3*c*; electronic supplementary material, table S5), for both the entire time (*p* = 0.002; electronic supplementary material, table S5), and for the 2000–2016 period (*p* = 0.00511; electronic supplementary material, table S5).

## 4. Discussion

Our results show that vascular plant assemblages that contain alien species are experiencing elevated rates of compositional rearrangement, most of which is explained by species replacement (i.e. turnover), compared to sites without aliens (figure 2; electronic supplementary material, table S4). Assemblages where aliens are present also exhibit higher levels of rank shift (figure 3). Rank shift is known to be an informative metric of biodiversity change [37]. Remarkably, these changes occur even though alien species are typically rare in invaded assemblages (electronic supplementary material, figure S15). Although there are marked differences in temporal beta diversity in assemblages with and without alien species, these assemblages are comparable in terms of alpha diversity (figure 3). Both types of assemblage in our analysis have equivalent rates of temporal change in species richness (figure 2*b*). Put another way, we find no evidence of changes in nestedness between areas with and without aliens, but higher rates of beta diversity change in areas with aliens compared to areas without aliens. As such, aliens are associated with greater levels of compositional reorganization, as well as exhibiting elevated temporal beta diversity at the species level thus driving local extinction as well as changes in relative abundance [30]. However, we stress that this is a correlational study, and the structure of the data does not allow us to rule out the alternative hypothesis that communities that are invaded or not are not random sub-samples of all communities.

Our study contributes to advancing our knowledge on the impacts of aliens by highlighting the potential role of alien species in leading to accelerated compositional change. Targeted studies, including experimental plots, will help uncover the mechanisms involved. Indeed, previous work has shown that invasive pines (*Pinus elliottii*) in the Cerrado biodiversity hotspot can contribute to biotic homogenization [11], with the impacts manifested through shifts in the species abundance distribution occurring at different spatial scales [28]. In most of the assemblages evaluated here, alien species are rare (electronic supplementary material, figure S15), which likely corresponds to earlier stages of invasion. Despite this, we were able to detect higher rates of compositional change in areas where these species are present. We could expect that as invasions proceed and aliens become established and increase their abundances in the assemblages, more profound changes in species composition would emerge. For example, the Cerrado biodiversity hotspot has been invaded by *Urochloa decumbens* and *Melinis minutiflora*, two key aggressive invasive grasses [11,55,56]. These species are the most dominant ones in parts of the Cerrado (encompassing 30% or more of the total assemblage abundance) and therefore also potentially more capable of impacting the native vegetation.

Elevated rates of turnover are being seen in natural assemblages across the world [1,3]. Previous studies, including those using BioTIME data, have found that although there is no systematic net change in alpha diversity, there is a marked signal of compositional change over time [1,3]. One potential driver contributing to this pattern of biodiversity change is the presence of introduced (and invasive) species [1,3,33,34]. By separating sites with and without aliens, we have highlighted a pervasive effect linked with invasion: namely faster turnover rates in invaded sites. From a conservation perspective, this means that aliens may drive local extinctions. From an ecological perspective, there might be some environmental limits to alpha diversity, e.g. related to resource partitioning, which means that incoming alien species either lose out, or, if they establish, drive others to extinction.

Turnover is a natural component of community structure. Natural assemblages are not fixed entities but rather dynamic [57] systems. A key question in ecology and conservation science that remains to be answered is what an optimal (and desirable) level of turnover in natural assemblages would be. A recent study evaluated the trajectories of greater than 1800 plant species across various habitat types in Europe and showed that communities are increasingly comprised larger range species to the detriment of smaller range species [58]. The authors of the study [58] argue that the characteristics of the winner species are in line with characteristics of aliens (large-range and nutrient-demanding species). These trends could contribute to a reduction in assemblage distinctiveness.

Like other recent meta-analyses using BioTIME [1,3,59], our study has a spatial bias (figure 1). For instance, only 16 out of the 52 sampled assemblages are located in tropical (or subtropical) areas. Similarly, another data limitation, which is not exclusive to BioTIME, is that one of the most vulnerable areas to climate change—areas with high endemism (e.g. oceanic islands and mountain ecosystems), as well as ecosystems expected to expand due to climate change (e.g. drylands), are underrepresented in databases. There is also variation in study duration, which we have attempted to control by focusing on biodiversity change in a subset of the most recent years (2000–2016). Furthermore, we must bear in mind that the studies available in BioTIME were not designed to specifically test the effect or presence of alien species, and as such, there is no paired design of invaded versus uninvaded sites for the same assemblage available. As we could not control for the presence of other potential drivers leading to biodiversity change, we cannot rule out the hypothesis that invaded sites might have pre-existing conditions which allow for the presence of aliens.

Future work could explore other aspects of biodiversity change specifically testing the impacts of alien species, such as shifts in the phylogenetic and functional diversity of invaded assemblages. For instance, alien species have been found to reduce taxonomic and phylogenetic beta diversity, but not functional beta diversity [31].

Nonetheless, despite these caveats, we could detect a tangible signal of alien species on biodiversity change linked to alien species in these assemblages even though these species are mostly rare. This result emphasizes the importance of preventing further invasions and managing existing invasions. Our study also underlines the necessity of evaluating compositional change in the context of invasions and reinforces the finding that metrics of species richness (alpha diversity) alone provide only a very partial indication of biodiversity change [60].

## Data Availability

The datasets supporting this article are provided in the electronic supplementary material [61].
